# Frequency of cannabis and illicit opioid use among people who use drugs and report chronic pain: A longitudinal analysis

**DOI:** 10.1371/journal.pmed.1002967

**Published:** 2019-11-19

**Authors:** Stephanie Lake, Zach Walsh, Thomas Kerr, Ziva D. Cooper, Jane Buxton, Evan Wood, Mark A. Ware, M. J. Milloy

**Affiliations:** 1 British Columbia Centre on Substance Use, Vancouver, British Columbia, Canada; 2 School of Population and Public Health, University of British Columbia, Vancouver, British Columbia, Canada; 3 Department of Psychology, University of British Columbia, Kelowna, British Columbia, Canada; 4 Department of Medicine, University of British Columbia, Vancouver, British Columbia, Canada; 5 Department of Psychiatry and Biobehavioral Sciences, University of California, Los Angeles, Los Angeles, California, United States of America; 6 British Columbia Centre for Disease Control, Vancouver, British Columbia, Canada; 7 Department of Family Medicine, McGill University, Montreal, Québec, Canada; 8 Department of Anesthesia, McGill University, Montreal, Québec, Canada; Massachusetts General Hospital, UNITED STATES

## Abstract

**Background:**

Ecological research suggests that increased access to cannabis may facilitate reductions in opioid use and harms, and medical cannabis patients describe the substitution of opioids with cannabis for pain management. However, there is a lack of research using individual-level data to explore this question. We aimed to investigate the longitudinal association between frequency of cannabis use and illicit opioid use among people who use drugs (PWUD) experiencing chronic pain.

**Methods and findings:**

This study included data from people in 2 prospective cohorts of PWUD in Vancouver, Canada, who reported major or persistent pain from June 1, 2014, to December 1, 2017 (*n* = 1,152). We used descriptive statistics to examine reasons for cannabis use and a multivariable generalized linear mixed-effects model to estimate the relationship between daily (once or more per day) cannabis use and daily illicit opioid use. There were 424 (36.8%) women in the study, and the median age at baseline was 49.3 years (IQR 42.3–54.9). In total, 455 (40%) reported daily illicit opioid use, and 410 (36%) reported daily cannabis use during at least one 6-month follow-up period. The most commonly reported therapeutic reasons for cannabis use were pain (36%), sleep (35%), stress (31%), and nausea (30%). After adjusting for demographic characteristics, substance use, and health-related factors, daily cannabis use was associated with significantly lower odds of daily illicit opioid use (adjusted odds ratio 0.50, 95% CI 0.34–0.74, *p <* 0.001). Limitations of the study included self-reported measures of substance use and chronic pain, and a lack of data for cannabis preparations, dosages, and modes of administration.

**Conclusions:**

We observed an independent negative association between frequent cannabis use and frequent illicit opioid use among PWUD with chronic pain. These findings provide longitudinal observational evidence that cannabis may serve as an adjunct to or substitute for illicit opioid use among PWUD with chronic pain.

## Abstract 2

M-J Milloy and colleagues investigate use of cannabis and illicit opioids among people who use drugs reporting chronic pain.

## Author summary

**Why was this study done?:**

- High numbers of people who use (illicit) drugs (PWUD) experience chronic pain, and previous research shows that illicit use of opioids (e.g., heroin use, non-prescribed use of painkillers) is a common pain management strategy in this population.
- Previous research has suggested that some patients might substitute opioids (i.e., prescription painkillers) with cannabis (i.e., marijuana) to treat pain.
- Research into cannabis as a potential substitute for illicit opioids among PWUD is needed given the high risk of opioid overdose in this population.
- We conducted this study to understand if cannabis use is related to illicit opioid use among PWUD who report living with chronic pain in Vancouver, Canada, where cannabis is abundant and the rate of opioid overdose is at an all-time high.

**What did the researchers do and find?:**

- Using data from 2 large studies of PWUD in Vancouver, Canada, we analyzed information from 1,152 PWUD who were interviewed at least once and reported chronic pain at some point between June 2014 and December 2017.
- We used statistical modelling to estimate the odds of daily opioid use for (1) daily and (2) occasional users of cannabis relative to non-users of cannabis, holding other factors (e.g., sex, race, age, use of other drugs, pain severity) equal.
- For participants who reported cannabis use, we also analyzed their responses to a question about why they were using cannabis (e.g., for intoxication, for pain relief)
- We found that people who used cannabis every day had about 50% lower odds of using illicit opioids every day compared to cannabis non-users. People who reported occasional use of cannabis were not more or less likely than non-users to use illicit opioids on a daily basis. Daily cannabis users were more likely than occasional cannabis users to report a number of therapeutic uses of cannabis including for pain, nausea, and sleep.

**What do these findings mean?:**

- Although more experimental research (e.g., randomized controlled trial of cannabis coupled with low-dose opioids to treat chronic pain among PWUD) is needed, these findings suggest that some PWUD with pain might be using cannabis as a strategy to alleviate pain and/or reduce opioid use.

## Introduction

Opioid-related morbidity and mortality continue to rise across Canada and the United States [1,2]. In many regions, including Vancouver, Canada—where drug overdoses were declared a public health emergency in 2016—the emergence of synthetic opioids (e.g., fentanyl) in illicit drug markets has sparked an unprecedented surge in death [3]. The overdose crisis is also the culmination of shifting opioid usage trends (i.e., from initiating opioids via heroin to initiating with pharmaceutical opioids [4]) that can be traced back, in part, to the over-prescription of pharmaceutical opioids for chronic non-cancer pain [5].

Despite this trend of liberal opioid prescribing, certain marginalized populations experiencing high rates of pain, including people who use drugs (PWUD), lack access to adequate pain management through the healthcare system [6,7]. Under- or untreated pain in this population can promote higher-risk substance use, as patients may seek illicit opioids (i.e., unregulated heroin or counterfeit/diverted pharmaceutical opioids) to manage pain [6,7]. In Vancouver, this practice poses a particularly high risk of accidental overdose, as estimates show that almost 90% of drugs sold as heroin are contaminated with synthetic opioids, such as fentanyl [8]. Another less-examined pain self-management strategy among PWUD is the use of cannabis [9]. Unlike illicit opioids and illicit stimulants, the cannabis supply (unregulated or regulated) has not been contaminated with fentanyl, and cannabis is not known to pose a direct risk of fatal overdose [10]. As a result, cannabis has been embraced by some, including emerging community-based harm reduction initiatives in Vancouver, as a possible substitute for opioids in the non-medical management of pain and opioid withdrawal [11]. Further, clinical evidence supports the use of cannabis or cannabinoid-based medications for the treatment of certain types of chronic non-cancer pain (e.g., neuropathic pain) [12].

As more jurisdictions across North America introduce legal frameworks for medical or non-medical cannabis use, ecological studies have provided evidence to suggest that states providing access to legal cannabis experience population-level reductions in opioid use [13–17], opioid dependence [18,19], and fatal overdose [19–21]. However, these state-level trends do not necessarily represent changes within individuals [22], highlighting a critical need to conduct individual-level research to better understand whether cannabis use is associated with reduced use of opioids and risk of opioid-related harms, particularly among individuals with pain. Of particular interest is a possible opioid-sparing effect of cannabis, whereby a smaller dose of opioids provides equivalent analgesia to a larger dose when paired with cannabis. Although this effect has been identified in pre-clinical studies [23], much of the current research in humans is limited to patient reports of reductions in the use of prescription drugs (including opioids) as a result of cannabis use [24–34]. However, a recent study among patients on long-term prescription opioid therapy produced evidence to counter the narrative that cannabis use leads to meaningful reductions in opioid prescriptions or dose [35]. These divergent findings confirm an ongoing need to understand this complex issue. To date, there is a lack of research from real-world settings exploring the opioid-sparing potential of cannabis among high-risk individuals who may be engaging in frequent illicit opioid use to manage pain. We therefore sought to examine whether frequency of cannabis use was related to frequency of illicit opioid use among PWUD who report living with chronic pain in Vancouver, Canada, the setting of an ongoing opioid overdose crisis.

## Methods

### Study sample

Data for this study were derived from 2 ongoing open prospective cohort studies of PWUD in Vancouver, Canada. The Vancouver Injection Drug Users Study (VIDUS) consists of HIV-negative people who use injection drugs [36]. The AIDS Care Cohort to evaluate Exposure to Survival Services (ACCESS) consists of people living with HIV who use drugs [37]. The current study, nested within these cohorts, was designed as part of a larger doctoral research project (SL) examining cannabis use and access among PWUD in the context of changing cannabis policy and the ongoing opioid overdose crisis [38]. The analysis plan for this study is provided in S1 Text. This study is reported as per the Strengthening the Reporting of Observational Studies in Epidemiology (STROBE) guidelines for cohort studies (S1 Checklist).

Recruitment for the cohort studies has been ongoing since 1996 (VIDUS) and 2005 (ACCESS) through extensive street outreach in various areas across Vancouver’s downtown core, including the Downtown Eastside (DTES), a low-income neighbourhood with an open illicit drug market and widespread marginalization and criminalization. To be eligible for VIDUS, participants must report injecting drugs in the previous 30 days at enrolment. To be eligible for ACCESS, participants must report using an illicit drug (other than or in addition to cannabis, which was a controlled substance under Canadian law until October 17, 2018) in the previous 30 days at enrolment. For both cohorts, HIV serostatus is confirmed through serology. Other eligibility requirements include being aged 18 years or older, residing in the Metro Vancouver Regional District, and providing written informed consent. Aside from HIV-disease-specific assessments, all study instruments and follow-up procedures are harmonized between the 2 studies to facilitate combined data analysis and interpretation.

At study enrolment, participants complete an interviewer-administered baseline questionnaire. Every 6 months thereafter, participants are eligible to complete a follow-up questionnaire. The questionnaires elicit information on socio-demographic characteristics, lifetime (baseline) and past-6-month (baseline, follow-up) patterns of substance use, risk behaviours, healthcare utilization, social and structural exposures, and other health-related factors. Nurses collect blood samples for HIV testing (VIDUS) or HIV clinical monitoring (ACCESS) and hepatitis C virus serology, providing referrals to appropriate healthcare services as needed. Participants are provided a Can$40 honorarium for their participation at each study visit.

### Ethics statement

Ethics approval for this study was granted by the University of British Columbia/Providence Health Care Research Ethics Board (VIDUS: H14-01396; ACCESS: H05-50233). Written informed consent was obtained from all study participants.

### Measures

To examine the use of illicit opioids and cannabis for possible ad hoc management of pain among PWUD, we restricted the study sample to individuals experiencing major or persistent pain. Beginning in follow-up period 17 (i.e., June 2014), the following question was added to the study questionnaire: “In the last 6 months, have you had any major or persistent pain (other than minor headaches, sprains, etc.)?” We included all observations from participants beginning at the first follow-up interview in which they reported chronic pain. For example, a participant who responded “no” to the pain question at follow-up 17 and “yes” at follow-up 18 would be included beginning at follow-up 18. For the purpose of these analyses, this first follow-up period with a pain report is considered the “baseline” interview.

The outcome of interest was frequent use of illicit opioids, defined as reporting daily (once or more per day) non-medical use of heroin or pharmaceutical opioids (diverted, counterfeit, or not-as-prescribed use) by injection or non-injection (i.e., smoking, snorting, or oral administration) in the previous 6 months. This outcome was captured through 4 different multipart questions based on class of opioid (i.e., heroin and pharmaceutical opioids) and mode of administration (i.e., injection and non-injection). For example, at each 6-month period, injection heroin use was assessed through the question: “In the last 6 months, when you were using, which of the following injecting drugs did you use, and how often did you use them?” Respondents were provided a list of commonly injected drugs, including heroin, and were asked to estimate their average frequency of injection in the past 6 months according to the following classifications: <1/month, 1–3/month, 1/week, 2–3/week, ≥1/day. An identical question for non-injection drugs assessed the frequency of non-injection heroin use. Pharmaceutical opioid injection was assessed through the question “In the past 6 months, have you injected any of the following prescription opioids? If so, how often did you inject them?” Participants were provided a list of pharmaceutical opioids with corresponding pictures for ease of identification. The question was repeated for non-injection use of pharmaceutical opioids, and the frequency categories were identical to those listed above. Using frequency categorizations from these 4 questions, participants who endorsed past-6-month daily injection or non-injection of heroin or pharmaceutical opioids were coded as “1” for the outcome (i.e., daily illicit opioid use) for that follow-up period. The main independent variable was cannabis use, captured through the question “In the last 6 months, have you used marijuana (either medical or non-medical) for any reason (e.g., to treat a medical condition or for a non-medical reason, like getting high)?” Those who responded “yes” were also asked to estimate their average past-6-month frequency of use according to the frequency categories described above. Frequency was further categorized as “daily” (i.e., ≥1/day), “occasional” (i.e., <1/month, 1–3/month, 1/week, 2–3/week), and “none” (no cannabis use; reference category). Sections of the questionnaire used for sample restriction and main variable building are provided in S2 Text.

We also considered several socio-demographic, substance use, and health-related factors with the potential to confound the association between cannabis use and illicit opioid use. Secondary socio-demographic variables included in this analysis were sex (male versus female), race (white versus other), age (in years), employment (yes versus no), incarceration (yes versus no), homelessness (yes versus no), and residence in the DTES neighbourhood (yes versus no). We considered the following substance use patterns: daily crack or cocaine use (yes versus no), daily methamphetamine use (yes versus no), and daily alcohol consumption (yes versus no). Health-related factors that were hypothesized to bias the association between cannabis and opioid use were enrolment in opioid agonist treatment (i.e., methadone or buprenorphine/naloxone; yes versus no), HIV serostatus (HIV-positive versus HIV-negative), prescription for pain (including prescription opioids; yes versus no), and average past-week pain level (mild–moderate, severe, or none). The pain variable was self-reported using a pain scale ranging from 0 (no pain) to 10 (worse possible pain). We used 3 as the cut-point for mild–moderate pain and 7 as the cut-point for moderate–severe pain. Although there is no universal standard for pain categorization, these cut-points are common and have been validated in other pain populations [39]. Due to low cell count for mild pain (scores 1–3), we collapsed this variable with moderate pain (4–6) to create the mild–moderate category. With the exception of sex and race, all variables are time-updated and refer to behaviours and exposures in the 6-month period preceding the interview. All variables except HIV status were derived through self-report. As data for the present study were derived from 2 large cohort studies with broader objectives of monitoring changing health and substance use patterns in the community, the study participants and interviewers were blinded to the objective of this particular study.

### Statistical analysis

We explored differences in characteristics at baseline according to daily cannabis use status (versus occasional/none) using chi-squared tests for categorical variables and Wilcoxon rank-sum tests for continuous variables. Then, we estimated bivariable associations between each independent variable and the outcome, daily illicit opioid use, using generalized linear mixed-effects models (GLMMs) with a logit-link function to account for repeated measures within individuals over time. Next, we built a multivariable GLMM to estimate the adjusted association between frequency of cannabis use and illicit opioid use. We used the least absolute shrinkage and selection operator (LASSO) approach to determine which variables to include in the multivariable model. This method uses a tuning parameter to penalize the model based on the absolute value of the magnitude of coefficients (i.e., L1 regularization), shrinking some coefficients down to 0 (i.e., indicating their removal from the multivariable GLMM). Four-fold cross-validation was used to determine the optimal value of the tuning parameter. GLMMs were estimated using complete cases (98.6%–100% of observations for bivariable estimates; 99.0% of observations for multivariable estimates).

In the most recent follow-up period (June 1, 2017, to December 1, 2017), participants who reported any cannabis use in the previous 6-month period were eligible for the follow-up question: “Why did you use it?” Respondents could select multiple options from a list of answers or offer an alternative reason under “Other”. These data were analyzed descriptively, and differences between at least daily and less than daily cannabis users were analyzed using a chi-squared test, or Fisher’s test for small cell counts.

All analyses were performed in RStudio (version 1.1.456; R Foundation for Statistical Computing, Vienna, Austria). All *p*-values are 2-sided.

## Results

Between June 1, 2014, and December 1, 2017, 1,489 participants completed at least 1 study visit and were considered potentially eligible for these analyses. Of them, 13 participants were removed due to missing data on the fixed variable for race (*n* = 9), no response to the pain question (*n* = 1), or multiple interviews during a single follow-up period (*n* = 3). Of the remaining 1,476 participants, 1,152 (78.0%) reported major or persistent pain during at least one 6-month follow-up period and were included in this analysis. We considered all observations from these individuals beginning from the first report of chronic pain, yielding 5,350 study observations, equal to 2,676.5 person-years of observation. There were 424 (36.8%) female participants in the analytic sample, and the median age at the earliest analytic interview was 49.3 years (IQR 42.3–54.9).

Over the study period, a total of 410 (35.6%) respondents reported daily and 557 (48.4%) reported occasional cannabis use throughout at least 1 of the 6-month follow-up periods; 455 (39.5%) reported daily illicit opioid use throughout at least 1 of the 6-month follow-up periods. At baseline (i.e., the first interview in which chronic pain was reported), 583 (50.6%) participants were using cannabis either occasionally (*n* = 322; 28.0%) or daily (*n* = 261; 22.7%), and 269 (23.4%) were using illicit opioids daily. At baseline, 693 (60.2%) participants self-reported a lifetime chronic pain diagnosis including bone, mechanical, or compressive pain (*n* = 347; 50.1%); inflammatory pain (*n* = 338; 48.8%); neuropathic pain (*n* = 129; 18.6%); muscle pain (*n* = 54; 7.8%); headaches/migraines (*n* = 41; 5.9%); and other pain (*n* = 53; 7.6%).

Table 1 provides a summary of baseline characteristics of the sample stratified by daily cannabis use status (yes versus no). Daily cannabis use at baseline was significantly more common among men (odds ratio [OR] 1.76, 95% 95% CI 1.30–2.38, *p <* 0.001) and significantly less common among those who used illicit opioids daily (OR 0.54, 95% CI 0.37–0.77, *p <* 0.001).

**Table 1:** Baseline characteristics of 1,152 people who use drugs with chronic pain, stratified by daily cannabis use.

Characteristic	Daily cannabis use		Odds ratio (95% CI)	*p*-Value
	Yes, *n* = 261 (22.7%)	No, *n* = 891 (77.3%)		
Age, years, median (IQR)	49.0 (42.0–54.5)	49.6 (42.4–48.5)	0.99 (0.98–1.01)	0.391
Sex				
Male	190 (72.8)	538 (60.4)	1.76 (1.30–2.38)	<0.001
Female	71 (27.2)	353 (39.6)	1.00	
Race				
White	140 (53.6)	491 (55.1)	0.94 (0.71–1.24)	0.728
Other	121 (46.4)	400 (44.9)	1.00	
Employment*				
Yes	74 (28.4)	206 (23.1)	1.32 (0.96–1.80)	0.099
No	187 (71.6)	685 (76.9)	1.00	
Incarceration*				
Yes	15 (5.8)	49 (5.5)	1.05 (0.58–1.91)	0.985
No	244 (94.2)	840 (94.5)	1.00	
Downtown Eastside residency*				
Yes	141 (54.0)	521 (58.5)	0.83 (0.63–1.10)	0.227
No	120 (46.0)	370 (41.5)	1.00	
Homelessness*				
Yes	31 (11.9)	148 (16.3)	0.68 (0.45–1.03)	0.066
No	229 (88.1)	742 (83.4)	1.00	
Opioid agonist treatment*				
Yes	129 (49.4)	470 (53.3)	0.86 (0.65–1.13)	0.304
No	132 (50.6)	412 (46.7)	1.00	
Crack/cocaine use*				
At least daily	43 (16.5)	142 (16.0)	1.04 (0.72–1.51)	0.916
Less than daily	218 (83.5)	748 (84.0)	1.00	
Methamphetamine use*				
At least daily	22 (8.4)	102 (11.5)	0.71 (0.44–1.15)	0.202
Less than daily	239 (91.6)	788 (88.6)	1.00	
Alcohol use*				
At least daily	28 (10.9)	83 (9.4)	1.18 (0.75–1.86)	0.467
Less than daily	229 (89.1)	803 (90.6)	1.00	
Illicit opioid use*				
At least daily	40 (15.3)	229 (25.7)	0.52 (0.36–0.76)	<0.001
Less than daily	221 (84.7)	662 (74.3)	1.00	
HIV status				
HIV+	112 (42.9)	408 (45.8)	0.89 (0.67–1.18)	0.452
HIV−	149 (57.1)	483 (54.2)	1.00	
Prescription for pain*				
Yes	139 (54.1)	413 (47.3)	1.31 (0.99–1.74)	0.064
No	118 (45.9)	461 (52.7)	1.00	
Average past-week pain level				
Severe	101 (38.5)	330 (37.0)	1.24 (0.72–2.15)	0.525
Mild–moderate	139 (53.4)	474 (53.2)	1.19 (0.69–2.03)	0.618
None	19 (7.3)	77 (8.7)	1.00	

Data are *n* (percent) unless otherwise indicated. Cells for each variable might not add up to the column total, as participants can refuse to answer questions.

*Refers to exposures/behaviours in the previous 6 months.

In bivariable longitudinal analyses (Table 2), daily cannabis use was significantly and negatively associated with daily illicit opioid use (OR 0.60, 95% CI 0.40–0.90, *p =* 0.013). Other factors that were negatively associated with daily illicit opioid use in crude analyses were age (OR 0.90 per year older, 95% CI 0.88–0.92, *p <* 0.001), employment (OR 0.73, 95% CI 0.54–0.99, *p =* 0.044), HIV seropositivity (OR 0.41, 95% CI 0.26–0.65, *p <* 0.001), and having a prescription for pain medication (OR 0.67, 95% CI 0.51–0.88, *p =* 0.004). Significant positive associations with daily illicit opioid use were detected for DTES residency (OR 2.71, 95% CI 1.99–3.69, *p <* 0.001), homelessness (OR 2.95, 95% CI 2.06–4.20, *p <* 0.001), incarceration (OR 2.00, 95% CI 1.16–3.46, *p =* 0.013), daily crack or cocaine use (OR 2.77, 95% CI 1.94–3.96, *p <* 0.001), daily methamphetamine use (OR 6.63, 95% CI 4.31–10.19, *p <* 0.001), and pain level (OR 1.33, 95% CI 1.00–1.76, *p =* 0.046, for mild–moderate pain; OR 1.75, 95% CI 1.28–2.38, *p <* 0.001, for severe pain). As shown in Table 2, after adjustment for confounders, the odds of daily illicit opioid use were significantly lower during periods of daily cannabis use (adjusted OR 0.50, 95% CI 0.33–0.74, *p <* 0.001), but not during periods of occasional use (adjusted OR 0.94, 95% CI 0.69–1.27, *p =* 0.682), relative to periods of no cannabis use.

**Table 2:** Unadjusted and adjusted generalized linear mixed-effects models of factors associated with daily illicit opioid use among 1,152 people who use drugs with chronic pain in Vancouver, Canada.

Characteristic	Unadjusted		Adjusted	
	OR (95% CI)	*p*-Value	OR (95% CI)	*p*-Value
Cannabis use*				
Occasional versus none	1.04 (0.77–1.40)	0.818	0.94 (0.69–1.27)	0.682
Daily versus none	0.60 (0.40–0.90)	0.013	0.50 (0.33–0.74)	<0.001
Sex				
Male versus female	0.65 (0.41–1.03)	0.067		
Age				
Per year older	0.90 (0.88–0.92)	<0.001	0.92 (0.90–0.94)	<0.001
Race				
White versus other	0.98 (0.62–1.54)	0.920		
Follow-up period				
Per 6-month interval	0.99 (0.94–1.05)	0.767		
Downtown Eastside residency*				
Yes versus no	2.71 (1.99–3.69)	<0.001	2.12 (1.54–2.90)	<0.001
Homelessness*				
Yes versus no	2.95 (2.07–4.21)	<0.001	1.91 (1.33–2.73)	<0.001
Incarceration*				
Yes versus no	2.00 (1.16–3.46)	0.013	1.27 (0.73–2.22)	0.393
Employment*				
Yes versus no	0.73 (0.54–0.99)	0.044	0.79 (0.58–1.07)	0.134
Opioid agonist therapy*				
Yes versus no	0.90 (0.66–1.22)	0.495		
Daily alcohol consumption*				
Yes versus no	0.91 (0.57–1.44)	0.673		
Daily crack/cocaine use*				
Yes versus no	2.77 (1.94–3.96)	<0.001	2.74 (1.93–3.90)	<0.001
Daily methamphetamine use*				
Yes versus no	6.63 (4.31–10.19)	<0.001	4.60 (3.02–7.02)	<0.001
HIV serostatus				
Positive versus negative	0.41 (0.26–0.65)	<0.001	0.48 (0.32–0.74)	<0.001
Pain prescription*				
Yes versus no	0.67 (0.51–0.88)	0.004	0.86 (0.65–1.13)	0.274
Average past-week pain rate				
Mild–moderate versus none	1.33 (1.00–1.75)	0.046		
Severe versus none	1.75 (1.29–2.37)	<0.001		

*Refers to exposures/behaviours in the previous 6 months.

**Fig 1:**
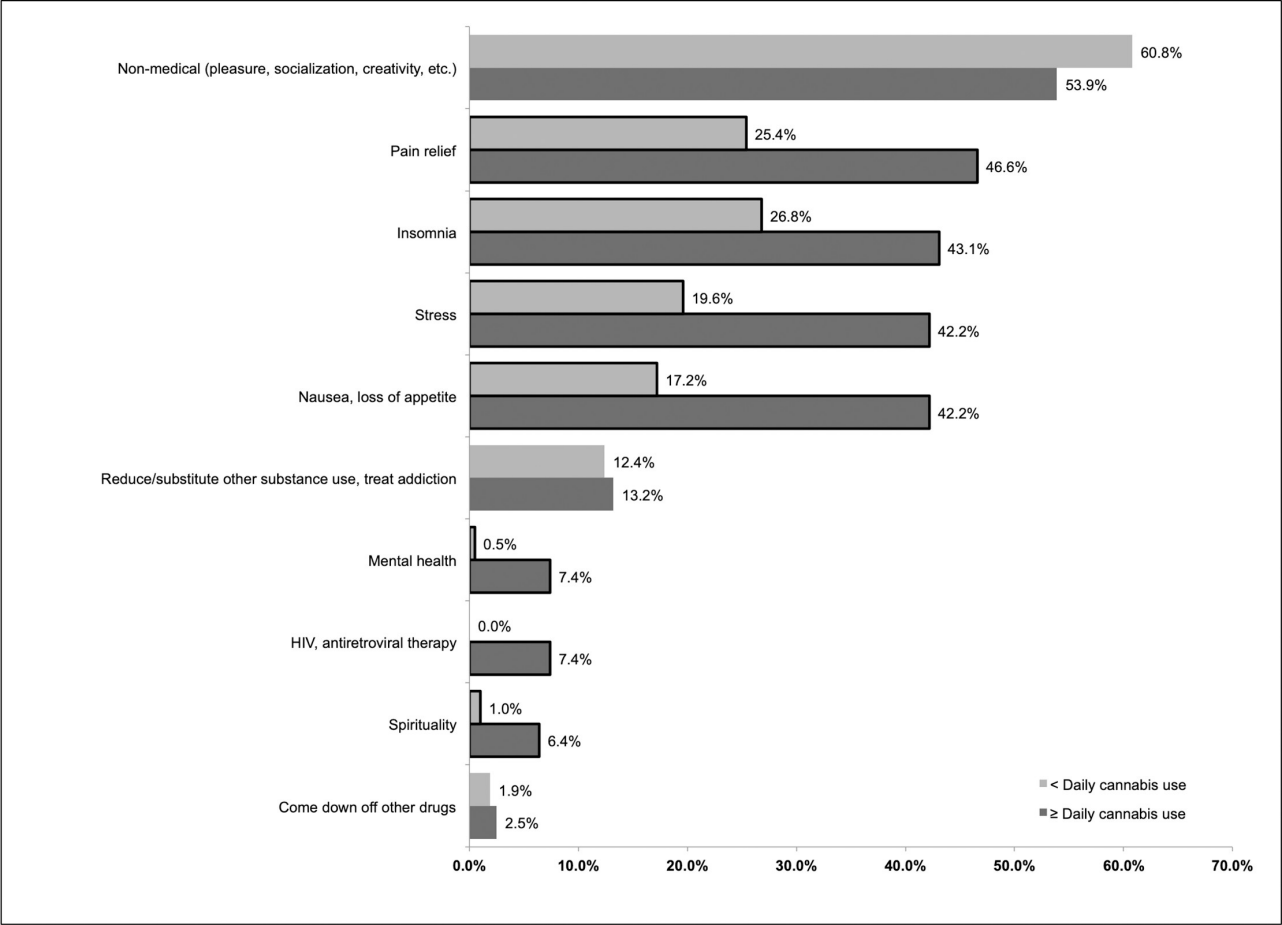
Self-reported reasons for cannabis use among daily (*n* = 204) and occasional (*n* = 210) cannabis-using people who use drugs with chronic pain, June–December 2017. Borders indicate chi-squared or Fisher’s test *p <* 0.05 (Fisher’s test used for mental health and HIV comparisons).

Of the 414 daily and occasional cannabis users who were interviewed from June 1, 2017, to December 1, 2017, the most commonly reported motivations for use were for recreation (i.e., to get high, socialize; *n* = 237; 57.2%), to manage pain (*n* = 148; 35.7%), to aid with sleep (*n* = 144; 34.8%), to manage stress (*n* = 127; 30.7%), to treat nausea or loss of appetite (*n* = 123; 29.7%), and to reduce the use of other substances/treat addiction or withdrawal (*n* = 53; 12.8%). Self-reporting pain, insomnia, stress, nausea, mental health, HIV, and spirituality as reasons for use were all significantly more common for daily cannabis users relative to occasional users (*p <* 0.05; Fig 1).

## Discussion

In this longitudinal study examining patterns of past-6-month frequency of cannabis and illicit opioid use, we found that the odds of daily illicit opioid use were lower (by about half) among those who reported daily cannabis use compared to those who reported no cannabis use. However, we observed no significant association between occasional cannabis use and daily opioid use, suggesting that there may be an intentional therapeutic element associated with frequent cannabis use. This is supported by cross-sectional data from the sample in which certain reasons for cannabis use were observed to differ according to cannabis use frequency. Specifically, daily users reported more therapeutic motivations for cannabis use (including to address pain, stress, nausea, mental health, or symptoms of HIV or antiretroviral therapy, or to improve sleep) than occasional users, and non-medical motivations—although common among all users—were not more likely to be reported by daily users. Together, our findings suggest that PWUD experiencing pain might be using cannabis as an ad hoc (i.e., improvised, self-directed) strategy to reduce the frequency of opioid use.

A recent study analyzed longitudinal data from a large US national health survey and found that cannabis use increases, rather than decreases, the risk of future non-medical prescription opioid use in the general population [40], providing important evidence to challenge the hypothesis that increasing access to cannabis facilitates reductions in opioid use. The findings of our study reveal a contrasting relationship between cannabis use and frequency of opioid use, possibly due to inherent differences in the sampled populations and their motivations for using cannabis. Within the current study population, poly-substance use is the norm; HIV and related comorbidities are common; and pain management through prescribed opioids is often denied, increasing the likelihood of non-medical opioid use for a medical condition [6,9]. Furthermore, our study is largely focused on this relationship in the context of pain (i.e., by examining individuals with self-reported pain and accounting for intensity of pain). Our findings align more closely with those of a recent study conducted among HIV-positive patients living with chronic pain, in which the authors found that patients who reported past-month cannabis use were significantly less likely to be taking prescribed opioids [41]. While this finding could have resulted from prescription denial associated with the use of cannabis (or any illicit drug), we show that daily cannabis users in this setting were slightly *more* likely to have been prescribed a pain medication at baseline, and adjusting for this factor in a longitudinal multivariable model did not negate the significant negative association of frequent cannabis use with frequent illicit opioid use.

The idea of cannabis as an adjunct to, or substitute for, opioids in the management of chronic pain has recently earned more serious consideration among some clinicians and scientists. A growing number of studies involving patients who use cannabis to manage pain demonstrate reductions in the use of prescription analgesics alongside favourable pain management outcomes [24–33]. For example, Boehnke et al. found that chronic pain patients reported a 64% mean reduction in the use of prescription opioids after initiating cannabis, alongside a 45% mean increase in self-reported quality of life [29]. Degenhardt et al. found that, in a cohort of Australian patients on prescribed opioids for chronic pain, those using cannabis for pain relief (6% of patients at baseline) reported better analgesia from adjunctive cannabis use (70% average pain reduction) than opioid use alone (50% average reduction) [33]. However, more recent high-quality research has presented findings to question this narrative. For example, in the 4-year follow-up analysis of the above Australian cohort of pain patients, no significant temporal associations were observed between cannabis use (occasional or frequent) and a number of outcomes including prescribed opioid dose, pain severity, opioid discontinuation, and pain interference [35]. Thus, several other explanations for our current results, aside from an opioid-sparing effect, are worthy of consideration.

We chose to include individuals with chronic pain regardless of their opioid use status to avoid exclusion of individuals who may have already ceased illicit opioid use at baseline, as these individuals may reflect an important subsample of those already engaged in cannabis substitution. On the other hand, there may be important characteristics, unrelated to pain, among regular cannabis users in this study that predispose them to engage in less frequent or no illicit opioid use at the outset. We attempted to measure and control for these factors, but we cannot rule out the possibility of a spurious connection. For example, individuals in this cohort who are consuming cannabis daily for therapeutic purposes may simply possess greater self-efficacy to manage health problems and control their opioid use [42]. However, it is notable that our finding is in line with a previous study demonstrating that cannabis use correlates with lower frequency of illicit opioid use among a sample of people who inject drugs in California, all of whom used illicit opioids [43]. Our study builds on this work by addressing chronic pain, obtaining detailed information on motivations for cannabis use, and examining longitudinal patterns.

We observed that daily cannabis users endorsed intentional use of cannabis for a range of therapeutic purposes that may influence pain and pain interference. After pain, insomnia (43%) and stress (42%) were the second and third most commonly reported motivations for therapeutic cannabis use among daily cannabis users. The inability to fall asleep and the inability stay asleep are common symptoms of pain-causing conditions [44], and experiencing these symptoms increases the likelihood of opioid misuse among chronic pain patients [45]. The relationship between sleep deprivation and pain is thought to be bidirectional [44,46], suggesting that improved sleep management may improve pain outcomes. Similarly, psychological stress (particularly in developmental years) is a well-established predictor of chronic pain [47] and is also likely to result from chronic pain. Thus, another possible explanation for our finding is that cannabis use substitutes for certain higher-risk substance use practices in addressing these pain-associated issues without necessarily addressing the pain itself.

Notably, our findings are consistent with emerging knowledge of the form and function of the human endocannabinoid and opioid receptor systems. The endogenous cannabinoid system, consisting of receptors (cannabinoid type 1 [CB1] and type 2 [CB2]) and modulators (the endocannabinoids anandamide and 2-arachidonoylglycerol), is involved in key pain processing pathways [48]. The co-localization of endocannabinoid and μ-opioid receptors in brain and spinal regions involved in antinociception [49], and the modification of one system’s nociceptive response via modulation of the other [50,51], has raised the possibility that the phytocannabinoid tetrahydrocannabinol (THC) might interact synergistically with opioids to improve pain management. A recent systematic review and meta-analysis found strong evidence of an opioid-sparing effect for cannabis in animal pain models, but little evidence from 9 studies in humans [23]. However, the authors of the meta-analysis identified several important limitations potentially preventing these studies in humans from detecting an effect, including low sample sizes, single doses, sub-therapeutic opioid doses, and lack of placebo [23]. Since then, Cooper and colleagues have published the results of a double-blind, placebo-controlled, within-subject study among humans in which they found that pain threshold and tolerance were improved significantly when a non-analgesic dose of an opioid was co-administered with a non-analgesic dose of cannabis [52]. Suggestive of a synergistic effect, these findings provide evidence for cannabis’s potential to lower the opioid dose needed to achieve pain relief [52].

Finally, there is pre-clinical and pilot clinical research to suggest that cannabinoids, particularly cannabidiol (CBD), may play a role in reducing heroin cue-induced anxiety and cravings [53] and symptoms of withdrawal [54]. Although preliminary, this research supports the idea that cannabis may also be used to stabilize individuals undergoing opioid withdrawal, as an adjunct to prescribed opioids to manage opioid use disorder, or as a harm reduction strategy. Although this evidence extends beyond chronic pain patients, it warrants consideration here given the shared history of illicit substance use amongst the study sample. It is not clear what role harm reduction or treatment motivations may have played in the current study since daily and occasional users did not differ significantly in reporting cannabis use as a strategy to reduce or treat other substance use. The phenomenon of using cannabis as a tool to reduce frequency of opioid injection has been highlighted through qualitative work in other settings [55], but further research is needed to determine whether this pattern is widespread enough to produce an observable effect. Clinical trials that can randomize participants to a cannabis intervention will be critical for establishing the effectiveness of cannabis both for pain management and as an adjunctive therapy for the management of opioid use disorder. Such trials would begin to shed light on whether the current finding could be causal, what the underlying mechanisms might be, and how to optimize cannabis-based interventions in clinical or community settings.

There are several important limitations to this study that should be taken into consideration. First, the cohorts are not random samples of PWUD, limiting the ability to generalize these findings to the entire community or to other settings. The older median age of the sample should especially be taken into consideration when interpreting these findings against those from other settings. Second, as discussed above, we cannot rule out the possibility of residual confounding. Third, aside from HIV serostatus, we relied on self-report for all variables, including substance use patterns. Previous work shows PWUD self-report to be reliable and valid against biochemical verification [56], and we have no reason to suspect that responses about the outcome would differ by cannabis use status, especially since this study was nested within a much larger cohort study on general substance use and health patterns within the community. Major or persistent pain, which qualified respondents for inclusion in this study, was also self-reported. Our definition for chronic pain is likely to be more sensitive than other assessments of chronic pain (e.g., clinical diagnoses or assessments that capture length of time with pain). Although more than half (60%) of the sample reported ever having been diagnosed with a pain condition, it is possible that some of the included respondents would not have met criteria for a formal chronic pain diagnosis. Finally, we did not collect information on the type of cannabis, mode of administration, cannabinoid content (e.g., percent THC:percent CBD), or dose during the study period. Future research will need to address these gaps to provide a more detailed picture of the instrumental use of cannabis for pain and other health concerns among PWUD.

## Conclusions

In conclusion, we found evidence to suggest that frequent use of cannabis may serve as an adjunct to or substitute for illicit opioid use among PWUD with chronic pain in Vancouver. The findings of this study have implications for healthcare and harm reduction service providers. In chronic pain patients with complex socio-structural and substance use backgrounds, cannabis may be used as a means of treating health problems or reducing substance-related harm. In the context of the current opioid crisis and the recent rollout of a national regulatory framework for cannabis use in Canada, frequent use of cannabis among PWUD with pain may play an important role in preventing or substituting frequent illicit opioid use. PWUD describe a wide range of motivations for cannabis use, some of which may have stronger implications in the treatment of pain and opioid use disorder. Patient–physician discussions of these motivations may aid in the development of a treatment plan that minimizes the likelihood of high-risk pain management strategies, yet there remains a clear need for further training and guidance specific to medical cannabis use for pain management [57].

## Supporting information

S1 Checklist STROBE checklist. (DOC)

S1 Text Study protocol. (DOC)

S2 Text Questionnaire sections for study’s main variables. (DOCX)

## Abbreviations

ACCESS: AIDS Care Cohort to evaluate Exposure to Survival Services
CBD: cannabidiol
DTES: Downtown Eastside
GLMM: generalized linear mixed-effects model
OR: odds ratio
PWUD: people who use illicit drugs
THC: tetrahydrocannabinol
VIDUS: Vancouver Injection Drug Users Study
