# Supplementary material for: Frequency of cannabis and illicit opioid use among people who use drugs and report chronic pain: A longitudinal analysis
Source: PLoS Med. 2019 Nov 19;16(11):e1002967. doi: 10.1371/journal.pmed.1002967 (PMC6863529; doi:10.1371/journal.pmed.1002967)
Supplement: S1 Text — (DOC) [file pmed.1002967.s002.doc]

**STATISTICAL SUPPORT FOR A BCCSU STUDY**

**Date of request**: August 9, 2018

**Name(s)**: Stephanie Lake (M-J Milloy)

**E-mail address(es)**: stephanie.lake@bccsu.ubc.ca

**Priority (indicate high upon PI’s approval)**: Normal

**Cohort(s)**: ACCESS, VIDUS

**Study Title**: Cannabis, opioids, morbidity among people who use illicit drugs

Study Context (include objectives and hypotheses, approx. 50-100 words): This request is to update one of my PhD datasets with newer follow-ups (23 and 24 if available) and to add a few additional variables for the time period December 2005 – Current. The dataset I currently have is updated to FU 22 inclusive.

1) Please provide updated data (FU 23 – Current) for all of the variable indicated in the original data request (attached below).

**Statistical support for a UHRI (BCCSU) study**

**Date of request**: May 30, 2017

**Name(s)**: Stephanie Lake (M-J Milloy)

**E-mail address(es)**: slake@cfenet.ubc.ca

**Priority (indicate high upon PI’s approval)**: Normal

**Cohort(s)**: ACCESS, VIDUS

**Reference Questionnaire (include name and date approved)**: Combined baseline (June 1, 2013)

***NOTE: All variables of interest, specifically those that are less common, should be accompanied by the corresponding Question # from the reference questionnaire, for example: shared a pipe (D17), ever injected (F1). If the study is longitudinal, question #’s should refer to the baseline from which the analyses are to be conducted. Where possible, please include a table that lists question numbers, variables of interest, and how they should be defined (e.g., continuous, yes vs. no, <22 vs. ≥22, etc.). Please precede Nursing Questionnaire variables with “NQ”.***

**Study Title**: Cannabis, opioids, morbidity among people who use illicit drugs

Study Context (include objectives and hypotheses, approx. 50-100 words): This request is for a dataset that I will use for one of my PhD analyses under M-J’s supervision.

Study Period: December 2005 (start of ACCESS) – most recent F/U

Study Sample (include inclusion and exclusion criteria): None

**DATASET VARIABLES**

*****Please note this is for row-level (person-period) data*

**Dataset 1: From questionnaire**

| **Variable** | **Based on** | **Variable type** | **Definition/categorization** |
| --- | --- | --- | --- |
| Identifier |  |  | Different numeric code unique to each participant |
| Follow-up period | Date of interview | Ordinal |  |
| Cohort |  | Nominal |  |
| Age | Tracking | Continuous |  |
| Sex at birth | BL A1 | Binary | Male vs. Female |
| Ethnicity | BL | Nominal | Numeric code for each ethnicity |
| Homeless_L6M |  | Binary | Yes vs. No |
| DTES_L6M |  | Binary | Yes vs. No |
| Employment_L6M |  | Binary | Yes vs. No |
| Jail_L6M |  | Binary | Yes vs. no |
| Addiction treatment, type L6M | FU 19: R1 | Nominal | Numeric code for each (e.g., 1 = detox) |
| Alcohol, frequency L6M |  | Ordinal | Numeric code for each listed frequency |
| C. Meth non-injection, frequency L6M | D3 | Ordinal | Numeric code for each listed frequency |
| C. Meth injection, frequency L6M | FU 19: F34 | Ordinal | ‘’’’ |
| Cocaine non-injection, frequency L6M | D3 | Ordinal | ‘’’’ |
| Cocaine injection, frequency L6M | FU 19: F34 | Ordinal | ‘’’’ |
| Crack non-injection, frequency L6M | D3 | Ordinal | ‘’’’ |
| Crack injection, frequency L6M | FU 19: F34 | Ordinal | ‘’’’ |
| Speedball injection, frequency L6M | FU 19: F34 | Ordinal | ‘’’’ |
| Heroin non-injection, frequency L6M | D3 | Ordinal | ‘’’’ |
| Heroin injection, frequency L6M | FU 19: F34 | Ordinal | ‘’’’ |
| PO non-injection, frequency L6M | FU 19: D9 | Ordinal | ‘’’’ |
| PO non-injection, type – frequency L6M  ** This will include multiple variables | FU 19: D9 | Ordinal | Please create different variables for each non-injection P. opioid, with numeric frequency codes |
| PO injection, frequency L6M | FU 19: F35 | Ordinal | Numeric code for each listed frequency |
| PO injection, type – frequency L6M  ** This will include multiple variables | FU 19: F35 | Ordinal | Please create different variables for each injection P. opioid, with numeric frequency codes |
| Cannabis use, frequency | FU 19: D1 | Ordinal | Numeric code for each listed frequency |
| Cannabis use, reason(s) | FU 21: D3 | Nominal | Numeric code for each listed reason |
| Pain L6M | NFU 21: G2 | Binary | Yes vs. No |
| Pain diagnosis, type | NFU 21: G4 | Nominal | Numeric code for each possible answer |
| Pain intensity average past week | NFU 21: G5 | Continuous |  |
| Pain treatment medication | NFU G8 | Nominal | Numeric code for each listed medication |

**Background:**

The opioid crisis is one of Canada’s most pressing public health challenges. Exacerbated by the emergence of novel forms of high-potency opioids (such as fentanyl),^1^ rates of morbidity and mortality from opioids are at an all-time high^2^ and an official public health emergency in British Columbia.^3^ As described in my preliminary work,^4^ the problem is particularly grave in Vancouver, where poly-drug use involving heroin and prescription opioids is common and tightly linked with overdose and other morbidity. Accordingly, experts across North America are urgently calling for innovative evidence-based responses to this ongoing crisis.^5,6^

The Canadian government recently announced its intention to legalize the use of cannabis by adults. There is intriguing preliminary evidence that cannabis legalization may facilitate reduced opioid-related morbidity and mortality, most likely through substitution of opioids with cannabis. For example, jurisdictions in the US that enacted legal access to medical cannabis saw a 25% relative decline in opioid overdose deaths over the decade.^7^ Preliminary research among people who use drugs (PWUD) also demonstrates that cannabis may substitute high-risk opioid use.^8,9^ However, the possible role of cannabis in improving health outcomes among people who use opioids has yet to be systematically and longitudinally evaluated. Therefore, the proposed project aims to explore the impact of cannabis use and cannabis availability/access on opioid use patterns and a range of opioid-related outcomes among PWUD in Vancouver, Canada.

**References:** **1.** Jafari S, et al. Rising fentanyl-related overdose deaths in British Columbia. *Can J Addict*. 2015;6(1):4-6. **2.** Fischer B, et al. Correlations between population levels of prescription opioid use and prescription-opioid-related substance use treatment admissions in the USA and Canada since 2001. *Public Health*. 2012;126(9):749-51. **3.** The Canadian Press. B.C. first in Canada to declare public health emergency after fentanyl overdoses. Maclean's. April 14, 2016. Available from: <http://www.macleans.ca/news/canada/b-c-first-in-canada-to-declare-public-health-emergency-after-fentanyl-overdoses/>. **4.** Lake S. Illicit prescription opioid injection: Prevalence, characteristics, and health outcomes among people who inject drugs. Vancouver: University of British Columbia; 2015. **5.** Voon P. Opioids: A national crisis needs a federal response. The Globe and Mail. April 11, 2016. Available from: <http://www.theglobeandmail.com/opinion/opioids-a-national-crisis-needs-a-federal-response/article29574947/>. **6.** Kolodny A, et al. The prescription opioid and heroin crisis: A public health approach to an epidemic of addiction. *Annu Rev Public Health*. 2015;36:559-74. **7.** Bachhuber MA, et al. Medical cannabis laws and opioid analgesic overdose mortality in the United States, 1999-2010. *JAMA Intern Med*. 2014. 2016;17(6):739-44. **8** Kral AH, et al. Is cannabis use associated with less opioid use among people who inject drugs? *Drug Alcohol Depend*. 2015. **9.** Lau N, et al. A safer alternative: Cannabis substitution as harm reduction. *Drug Alcohol Rev*. 2015;34(6):654-9.

**Analysis Plan**

| **MAIN** | **Research question(s)** | Is frequency of cannabis use associated with frequency of opioid use among PWUD who report major / persistent pain? |
| --- | --- | --- |
|  | **Hypotheses** | Increased frequency of cannabis use will be associated with decreased frequency of opioid use |
|  | **Dataset(s)** | PhD Dataset 1 (above) |
|  | **Inclusion/exclusion criteria** | Restrict to observations on or after FU 17 (pain question added in FU 17 questionnaire). Include all VIDUS and ACCESS participants who report experiencing major or persistent pain in the L6M from the first FU period in which they reported pain. Exclude individuals with missing data on the outcome of interest (below). |
|  | **Missing data** | Remove missing observations, pending n (%) of missing observations. Consider other ways of handling missing data if ≥10% missing. |
|  | **Outcome variable** | Daily illicit opioid use, i.e., ≥ daily self-reported heroin or PO injection or non-injection in the L6M. |
|  | **Exposure variable** | Frequency of cannabis use, categorized into ≥ daily, occasional (self-reported < daily in the L6M), and no use in the L6M. |
|  | **Confounders / secondary covariates** | Potential confounders: age, sex, race, housing, employment, incarceration, area of residence, year of interview, other drug use (meth, crack/cocaine, alcohol), pain severity, prescribed pain medication, HIV status, |
|  | **Statistical methods** | Baseline comparisons (chi-square, mann whitney u), bivariable and multivariable longitudinal analyses (generalized linear mixed effects models) |
| **SECONDASRY** | **Research question(s)** | What are the common reasons for cannabis use among PWUD who report pain and cannabis use? |
|  | **Hypotheses** | No hypotheses – descriptive and exploratory analysis of preliminary (cross-sectional) data from the cohorts |
|  | **Dataset(s)** | PhD Dataset 1 (Above) |
|  | **Inclusion/exclusion criteria** | Include all respondents from main analysis who reported cannabis use in the FU 23. |
|  | **Outcome variable** | Daily cannabis use |
|  | **Exposure variable** | Binary categorizations (yes vs. no) for each reason for cannabis use (e.g., sleep; yes vs. no) |
|  | **Confounders / secondary covariates** | NA |
|  | **Statistical methods** | Descriptive and univariate (chi-square, Fisher’s) |
