# Supplementary material for: Frequency of cannabis and illicit opioid use among people who use drugs and report chronic pain: A longitudinal analysis
Source: PLoS Med. 2019 Nov 19;16(11):e1002967. doi: 10.1371/journal.pmed.1002967 (PMC6863529; doi:10.1371/journal.pmed.1002967)
Supplement: S2 Text — (DOCX) [file pmed.1002967.s003.docx]

170529


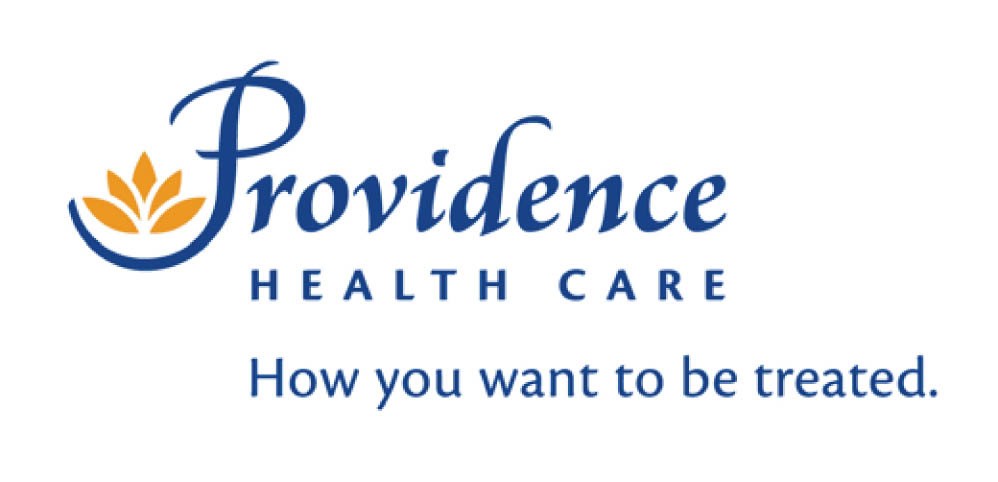

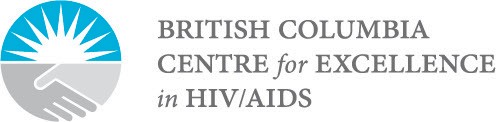

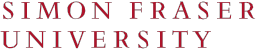

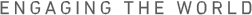

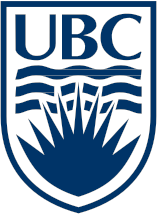


**Nurse’s Questionnaire**

**VDUS (VIDUS/ARYS) Follow-Up 23 / 18**

**VIDUS II Code**

H05-50234

**ARYS II Code**

H05-50160

**Participant Date of Birth:**

**Interviewer: Date of Interview:**

**Remove and shred this page after data cleaning.**

**VDUS (VIDUS/ARYS) Nurse’s Questionnaire: Follow-Up 23/18**

**G: Disability & Pain**

**G1.**

*F22*

❒

❒

❒

❒

❒

❒

❒

❒

❒

**G3.** Throughout our lives, most of us have had pain from time to time. In the **last 6 months**, have you had any **major or persistent pain** (other than minor headaches, sprains, etc.)?

❒ Yes ❒ No ***(Go to G7.)***

*F92d*

**VDUS (VIDUS/ARYS) Nurse’s Questionnaire: Follow-Up 23/18**

**G5.** Have you **ever** been diagnosed with a chronic pain condition? ***(Check all that apply.)***

*F93*

|  | ❒ | Neuropathic pain (e.g., nerve root compression, neuropathy, neuralgia, fibromyalgia) |
| --- | --- | --- |
|  | ❒ | Inflammatory pain (e.g., arthritis, infection, tissue injury, post-op pain) |
|  | ❒ | Muscle (i.e., soft tissue pain / myofascial pain syndrome) |
|  | ❒ | Bone / mechanical / compressive (e.g., low back, neck, visceral, strains, sprains, fractures, bone degeneration, osteoporosis, dislocation, compression by tumour / cyst / bony structure) |
|  | ❒ | Headache / migraine |
|  | ❒ | Other or unknown classification: |
|  | ❒ | **Never diagnosed with a chronic pain condition** |

**G6.** On a scale of zero to 10, with zero being no pain and 10 being the worst possible pain, how would you describe your **overall** pain …

*F96a, F96b, F96c, F96d*

**No** 0

| … **right now**? | … on **average**, over the  **past week**? | … at its **worst**, over the  **past week**? | … at its **least**, over the  **past week**? |
| --- | --- | --- | --- |
| ❒ | ❒ | ❒ | ❒ |
| ❒ | ❒ | ❒ | ❒ |
| ❒ | ❒ | ❒ | ❒ |
| ❒ | ❒ | ❒ | ❒ |
| ❒ | ❒ | ❒ | ❒ |
| ❒ | ❒ | ❒ | ❒ |
| ❒ | ❒ | ❒ | ❒ |
| ❒ | ❒ | ❒ | ❒ |
| ❒ | ❒ | ❒ | ❒ |
| ❒ | ❒ | ❒ | ❒ |
| ❒ | ❒ | ❒ | ❒ |

**pain**

1

2

3

4

**Moderate pain**

5

6

7

8

**Worst** 9

**possible**

**pain** 10

**G7.**

*F43d*

170615


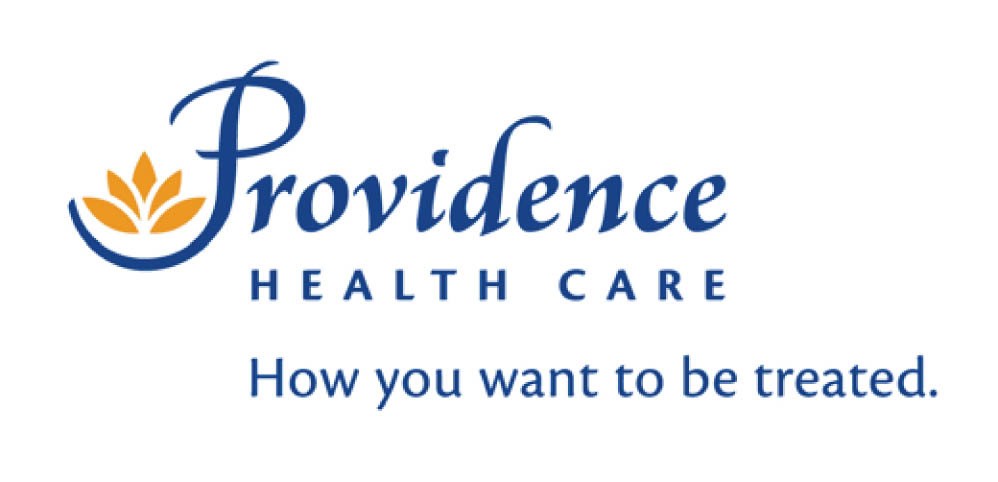

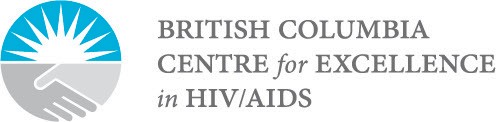

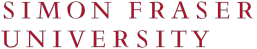

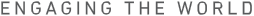

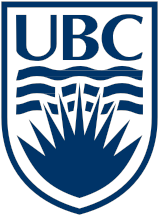


**Main Questionnaire**

**Follow-Up 23** / **18**

**VIDUS II / ACCESS FU 23**

**ARYS II FU 18**

**VIDUS II Code:**

H05-50234

**ARYS II Code:**

H04-50160

**ACCESS Code:**

H05-50233

**Participant Date of Birth:**

**Participant Last Study Visit:**

*(month / year)*

**Interviewer:**

**Date of Interview:**

***READ:***

Thank you for coming in today and contributing to this study. As we go through the interview together, please keep in mind that there are **no wrong answers**. It’s very important that you answer as honestly as you can. We rely on your information to **help create positive change** for people who use drugs.

We realize some of these questions are sensitive. If you do not want to answer a question, just let me know and we will move on. It is better for you to **refuse to answer** a question than to give a false answer. False answers affect the quality of our data and limit our ability to advocate for positive change.

We take your **privacy very seriously**. All the information that you provide will only be kept between you and me. We never report any individual information.

If there are any questions you don’t understand, please stop me and **ask for clarification**. The interview takes about an hour. If you need a break, let me know and we can stop for a short rest before we finish the interview.

**Remove and shred this page after data cleaning.**

**D: NON-INJECTION DRUG USE**

***READ:*** In this section, I am going to ask you about any drugs that you smoke, snort or ingest (swallow). In other words, I am going to ask about any drugs you use without a needle. This section also includes questions about alcohol.

*D45*

***READ:*** The next few questions are about marijuana use. We understand that people use marijuana for a variety of different reasons, many of which are positive. Through these questions we hope to better understand how and why you use marijuana.

**D3.** In the **last 6 months**, have you used marijuana (either medical or non-medical) for any reason (e.g., to treat a medical condition or for a non-medical reason, like getting high)?

Yes*****  No ***(Go to D9.)***

**D4.** How often did you use it?

*D44*

**Marijuana use frequency**

**If daily use**

**Duration of daily use over L6M *(in months)* Avg # of uses per**

**A B C D E < 1 1 to 3 3 to 6 > 6**

**day**

**D6.** Why did you use it?

| **L6M *(Check all that apply.)*** | **Most *(Check one only.)*** |  |
| --- | --- | --- |
|  |  | Pain relief, including MS, arthritis, etc. |
|  |  | Treat any mental health concern other than addiction |
|  |  | Help with sleep |
|  |  | Treat withdrawal |
|  |  | Substitute for other licit substance (e.g., alcohol, tobacco) |
|  |  | Substitute for illicit opioids |
|  |  | Substitute for illicit stimulants |
|  |  | Help with HIV medications or AIDS symptoms |
|  |  | Treat nausea / loss of appetite |
|  |  | “Get high,” recreation, quality of life, socializing |
|  |  | Spiritual |
|  |  | Creativity |
|  |  | Stress |
|  |  | Coming down off other drugs |
|  |  | Other: |

*D4a*

*D7*

*D2*

**D14.** In the **last 6 months** (that is, since ), when you were using, which of the following **non-** *D3*

**injecting** drugs did you use, and how often did you use them?

**Drug use frequency *(If daily use)* Duration of daily use**

**Non-injected drugs**

**over L6M *(in months)***

**A B C D E < 1 1 to 3 3 to 6 > 6**

Cocaine (non-injected)

Crack cocaine

Sleeping pills

Heroin (non-injected)

Crystal methamphetamine

Ecstasy / MDMA / Molly

Ketamine (Special K)

Benzos (benzodiazepines)

Fentanyl powder/pills

Prescription stimulant:

Gabapentin

Other: Other:

***READ:*** These are some new questions about prescription opioids. We are asking these questions because there are more and more people using prescription opioids in Canada. We want to better understand the changes in use of prescription opioids among people who use drugs.

**D15.** In the **last 6 months** (that is, since ), when you were using, which of the following **non- injection prescription opioids** did you use when they were not prescribed for you or that you took only for the experience or feeling they caused, and how often did you use them? ***(Show chart of prescription opioid drug names and pictures.)***

**No non-injection PO use in last 6 months *(Go to D17.)***

*D301*

**Non-injected POs**

**Drug use Frequency**

**If daily use**

**Duration of daily use**

**over L6M *(in months)* Average**

**# of uses**

**A B C D E < 1 1 – 3 3 – 6 > 6**

OxyNEO

OxyContin

Percocet (Percodan, other oxycodone)

Tylenol 3 (codeine)

Morphine (MS Contin, Avinza, Kadian, M-Eslon)

Dilaudid (hydromorphone hydrochloride)

Demerol (Darvon, meperidine, propoxyphene)

Methadose (Dolophine)

Suboxone

Fentanyl patch (Durgesic, Actiq)

Hydrocodone (Vicodin)

Talwin (pentazocine)

Tramadol

Other:

**per day**

Other:

**D16.** In the **last 6 months,** how often did you use (swallow, sniff/snort, smoke) any of these pills? ***(Show chart of prescription opioid drug names and pictures.)***

*D302*

**Non-Injection PO Use Frequency**

**If daily use**

**Duration of daily use over L6M *(in months)* Avg # of uses per**

**A B C D E < 1 1 to 3 3 to 6 > 6**

**day**

*D17*

**F21.** In the **last 6 months** (that is, since ), when you were using, which of the following drugs did you **inject,** and how often?

*F46*

**Non-PO Injection Drugs**

**Drug Use Frequency**

**If Daily Use**

**Duration of daily use**

**over L6M *(in months)* Average # of uses per day**

**A B C D E < 1 1 to 3 3 to 6 > 6**

Heroin alone

Cocaine alone

Heroin & cocaine

(“speedballs”)

Crystal methamphetamine

Heroin & crystal meth

(“goofballs”)

Crack cocaine

Fentanyl powder/pills

Benzos

Prescription stimulants:

Other drug:

**Have not injected any non-prescription drugs in the last 6 months *(Go to F22.)***

**F22.** In the **last 6 months** (that is, since ), have you **injected** any of the following **prescription opioids**? If so, how often did you use them? ***(Show chart of prescription opioid drug names and pictures.)***

*F461*

**Injection POs**

**Drug Use Frequency**

**If Daily Use**

**Duration of daily use**

**over L6M *(in months)* Average # of uses per day**

**A B C D E < 1 1 to 3 3 to 6 > 6**

OxyNEO

OxyContin

Percocet (Percodan, other

oxycodone)

Tylenol 3 (codeine)

Morphine (MS Contin, Avinza,

Kadian, M-Eslon)

Dilaudid (hydromorphone

hydrochloride)

Demerol (Darvon, meperidine,

propoxyphene)

Methadose (Dolophine)

Suboxone

Fentanyl patch (Durgesic, Actiq)

Hydrocodone (Vicodin)

Talwin (pentazocine)

Tramadol

Other:

Other:

__ **No injection PO use in the last 6 months *(Go to F24.)***

**F23.** In the **last 6 months,** how often did you **inject** any of these pills? ***(Show chart of prescription opioid drug names and pictures.)***

*F462*

**Injection PO Use Frequency**

**If daily use**

**Duration of daily use over L6M *(in months)***

**Avg # of uses per day**

**A B C D E < 1 1 to 3 3 to 6 > 6**

*F60*
